# A reverse chemical ecology approach to explore wood natural durability

**DOI:** 10.1111/1751-7915.13680

**Published:** 2021-02-17

Following the original publication of the Perrot *et al.* (2020), the author would like to amend the presentation of author names.

The first and last names of the authors should read as:

Thomas Perrot, Guillaume Salzet, Nadine Amusant, Jacques Beauchene, Philippe Gérardin, Stéphane Dumarçay, Rodnay Sormani, Mélanie Morel‐Rouhier, Eric Gelhaye

The authors would like to apologize for this error and any confusion it may have caused.
